# Nicotinic Acid Adenine Dinucleotide Phosphate (NAADP) and Endolysosomal Two-pore Channels Modulate Membrane Excitability and Stimulus-Secretion Coupling in Mouse Pancreatic β Cells*

**DOI:** 10.1074/jbc.M115.671248

**Published:** 2015-07-07

**Authors:** Abdelilah Arredouani, Margarida Ruas, Stephan C. Collins, Raman Parkesh, Frederick Clough, Toby Pillinger, George Coltart, Katja Rietdorf, Andrew Royle, Paul Johnson, Matthias Braun, Quan Zhang, William Sones, Kenju Shimomura, Anthony J. Morgan, Alexander M. Lewis, Kai-Ting Chuang, Ruth Tunn, Joaquin Gadea, Lydia Teboul, Paula M. Heister, Patricia W. Tynan, Elisa A. Bellomo, Guy A. Rutter, Patrik Rorsman, Grant C. Churchill, John Parrington, Antony Galione

**Affiliations:** From the ‡ Department of Pharmacology, University of Oxford, Mansfield Road, Oxford OX1 3QT, United Kingdom,; ‡‡ The Mary Lyon Centre, Medical Research Council Harwell, Oxfordshire OX11 0RD, United Kingdom,; the ¶ Nuffield Department of Surgery, John Radcliffe Hospital, Headley Way, Headington, Oxford OX3 9DU, United Kingdom,; the ** Henry Wellcome Centre for Gene Function, Department of Physiology, Anatomy, and Genetics, University of Oxford, South Parks Road, Oxford OX1 3QX, United Kingdom,; the § Centre des Sciences du Gout et de l'Alimentation, Equipe 5, 9E Boulevard Jeanne d'Arc 21000 Dijon, France,; the §§ Section of Cell Biology and Functional Genomics, Division of Diabetes, Endocrinology and Medicine, Imperial College London, Hammersmith Hospital, du Cane Road, London W12 0NN, United Kingdom, and; the ‖ The Oxford Centre for Diabetes, Endocrinology and Metabolism, Churchill Hospital, Oxford OX3 7LJ, United Kingdom

**Keywords:** diabetes, endosome, insulin, lysosome, nicotinic acid adenine dinucleotide phosphate (NAADP), TPC1, TPC2

## Abstract

Pancreatic β cells are electrically excitable and respond to elevated glucose concentrations with bursts of Ca^2+^ action potentials due to the activation of voltage-dependent Ca^2+^ channels (VDCCs), which leads to the exocytosis of insulin granules. We have examined the possible role of nicotinic acid adenine dinucleotide phosphate (NAADP)-mediated Ca^2+^ release from intracellular stores during stimulus-secretion coupling in primary mouse pancreatic β cells. NAADP-regulated Ca^2+^ release channels, likely two-pore channels (TPCs), have recently been shown to be a major mechanism for mobilizing Ca^2+^ from the endolysosomal system, resulting in localized Ca^2+^ signals. We show here that NAADP-mediated Ca^2+^ release from endolysosomal Ca^2+^ stores activates inward membrane currents and depolarizes the β cell to the threshold for VDCC activation and thereby contributes to glucose-evoked depolarization of the membrane potential during stimulus-response coupling. Selective pharmacological inhibition of NAADP-evoked Ca^2+^ release or genetic ablation of endolysosomal TPC1 or TPC2 channels attenuates glucose- and sulfonylurea-induced membrane currents, depolarization, cytoplasmic Ca^2+^ signals, and insulin secretion. Our findings implicate NAADP-evoked Ca^2+^ release from acidic Ca^2+^ storage organelles in stimulus-secretion coupling in β cells.

## Teaser

**Background:** TPCs are regulated by NAADP and other factors.

**Results:** NAADP-induced Ca^2+^ release from acidic stores evokes depolarizing currents in pancreatic β cells. Inhibition of NAADP signaling or TPC knock out attenuates Ca^2+^ signaling and insulin secretion.

**Conclusion:** NAADP-evoked Ca^2+^ release enhances β cell excitability and insulin secretion in response to glucose or sulfonylureas.

**Significance:** NAADP signaling pathways offer novel therapeutic targets for diabetes treatment.

## Introduction

Pancreatic β cells are electrically excitable, and in response to elevated blood glucose concentrations, oscillatory bursts of Ca^2+^ action potentials mediated by VDCCs^5^ are elicited. These drive cytosolic Ca^2+^ ([Ca^2+^]*_i_*) oscillations that, in turn, induce pulsatile insulin release (1), and defects in their generation may be associated with the loss of glucose homeostasis in type-2 diabetes (2). Glucose-evoked membrane depolarization results from the closure of ATP-dependent potassium (K_ATP_) channels, octameric complexes of sulfonylurea receptor 1 (SUR1) and inwardly rectifying Kir6.2 potassium channel subunits (3), and inactivating or activating mutations in the K_ATP_ channel (where the K_ATP_ channel is the ATP-dependent potassium channel) subunits lead to congenital hyperinsulinemia (4) or neonatal diabetes (5), respectively. However, K_ATP_ channel closure alone is not sufficient to depolarize the membrane to threshold, and activation of an additional depolarizing current has also been postulated (6, 7). The existence of an additional glucose-regulated membrane current in β cells is suggested by the finding that mice lacking functional K_ATP_ channels (8), like *Sur1* or *Kir6.2* knock-out mice), are not hypoglycemic, and islets from adult knock-out mice are still capable of responding to glucose with electrical activity, [Ca^2+^]*_i_* oscillations, and insulin secretion (9–11). The identity and regulation of this membrane conductance remain an enigma.

In contrast to the Ca^2+^ influx across the plasma membrane that plays a critical role in effecting insulin granule exocytosis, Ca^2+^ release from intracellular stores has been thought to play a modulatory rather than a triggering role in stimulus-secretion coupling in the pancreatic β cell. [Ca^2+^]*_i_* oscillations in response to glucose are modulated by the uptake and release of Ca^2+^ from endoplasmic reticulum (ER) Ca^2+^ stores (12) and also from acidic Ca^2+^ storage organelles (13). In addition, several incretins, such as glucagon-like peptide 1 and acetylcholine, are thought to enhance insulin secretion by mechanisms that are, in part, dependent on Ca^2+^ release from intracellular stores via intracellular messengers such as cAMP and inositol trisphosphate (IP_3_) (14, 15). However, recent studies have suggested that the newly discovered Ca^2+^-mobilizing messenger NAADP might play an important role in β cell Ca^2+^ signaling (16–24).

NAADP, the most potent of the Ca^2+^-mobilizing messengers described, has been shown to mediate local Ca^2+^-signaling events by releasing Ca^2+^ from acidic, endolysosomal Ca^2+^ stores in several vertebrate and invertebrate cells (25–27), and appears to be a critical trigger for many Ca^2+^-signaling events (26–28). The most prominent target Ca^2+^ release channels for NAADP have recently been identified as the two members of the endolysosomal two-pore channel family, TPC1 and TPC2 (29–37). Some studies report a lack of NAADP sensitivity in isolated lysosomes (23, 38), which may reflect technical issues, but also may be due in part to loss of NAADP binding to an accessory protein (39–42) forming part of a multiprotein signaling complex in endolysosomal membranes (27, 43–45). NAADP-induced Ca^2+^ release in MIN6 cells can be disrupted by the lysomotropic agent glycyl-l-phenylalanine-β-naphthylamide (GPN) or bafilomycin, which disrupts acidic store Ca^2+^ storage implicating lysosomally related organelles as the principal target for NAADP in these cells (19, 20, 23). In the pancreatic β cell line MIN6, and primary mouse β cells, glucose increases NAADP synthesis and hence intracellular levels (18, 20, 22), consistent with its role as an intracellular messenger. NAADP introduced into mouse pancreatic β cells via a patch pipette was found to evoke a series of oscillatory plasma membrane currents, which were blocked by the NAADP antagonist Ned-19 (21) and were abolished in pancreatic β cells prepared from *Tpcn2*^−/−^ mice (29). Furthermore, increasing concentrations of Ned-19 abolished glucose-evoked Ca^2+^ spiking in mouse pancreatic β cells, suggesting an important role for NAADP in stimulus-response coupling in these cells (21). This finding is consistent with our earlier study showing that prior desensitization of NAADP-sensitive Ca^2+^ release mechanisms block subsequent glucose-evoked Ca^2+^ signals in MIN6 cells (18).

Glucose (18) and glucagon like-peptide 1 (18, 20) have both been reported to increase β cell NAADP levels, effects that may be partially dependent on the ADP-ribosyl cyclase, CD38 (20, 22). At present, ADP-ribosyl cyclases, including CD38, are the only characterized enzymes that have been demonstrated to catalyze the synthesis of NAADP, using NADP and nicotinic acid as substrates by a base-exchange mechanism (46, 47). It has been suggested that glucose stimulation increases the internalization of CD38 involving cytoskeletal changes (22) with NAADP synthetic sites associated with acidic organelles (20). Furthermore, glucose-evoked Ca^2+^ signals and insulin secretion are impaired in mouse *Cd38*^−/−^ pancreatic β cells, and *Cd38*^−/−^ mice show glucose intolerance (48), and human CD38 autoantibodies and CD38 mutations have been shown to be associated with type-2 diabetes (49, 50). Recently, extracellular NAADP was found to be transported into mouse pancreatic β cells where it evoked Ca^2+^ release from acidic stores (24). Remarkably, intraperitoneal injections of NAADP were found to restore glucose-evoked insulin secretion in the *db/db* mouse model of type-2 diabetes and to ameliorate blood glucose regulation (24).

Here, we have used the cell-permeant analogue of NAADP, NAADP-AM (51), the selective cell-permeant NAADP antagonist Ned-19 (21), *Tpcn1*^−/−^ and *Tpcn2*^−/−^ mice (29), to explore a possible role for TPC-dependent NAADP-induced Ca^2+^ release from acidic stores in glucose-induced [Ca^2+^]*_i_* increases and insulin secretion in primary mouse β cells.

## Experimental Procedures

### 

#### 

##### Preparation of Islets of Langerhans and Islet β Cell Clusters

Islets of Langerhans were aseptically isolated by collagenase digestion of the pancreases of 8–10-week-old male mice of the following strains: CD1, *Tpcn2*^+/+^ and *Tpcn*2^−/−^ (29), *Tpcn1*^+/+^ and *Tpcn*1^−/−^ (52), with *Tpcn* mice in a B6;129 background. All mice were killed by cervical dislocation and age- and sex-matched (and for the latter two, background strain-matched). Except for the hormone release measurements (for which intact islets were used), clusters of islet β cells and single β cells were prepared by dispersing islets in a Ca^2+^-free medium and cultured on circular coverslips for 1–4 days in RPMI 1640 culture medium (GIBCO, Paisley, UK) containing 10% heat-inactivated fetal calf serum, 100 IU/ml penicillin, 100 μg/ml streptomycin, and 10 mm glucose.

##### [Ca^2+^]_i_ Measurements

Cultured clusters of islet cells were loaded with 1 μm Fura PE3-AM or Fura 2-AM (Teflabs, Austin, TX) for 60 min at 37 °C in a bicarbonate-buffered solution containing 10 mm glucose. The coverslip was then used as the bottom of a temperature-controlled perifusion chamber (Bioscience Tools, San Diego) mounted on the stage of an inverted microscope. The flow rate was 1.5 ml/min, and the temperature within the chamber was 37 °C. [Ca^2+^]*_i_* was measured at dual-wavelength (340 and 380 nm) excitation spectrofluorimetry, using a CCD camera (Photon Technologies International, Princeton, NJ) to capture the emitted fluorescence at 510 nm. When [Ca^2+^]*_i_* was simultaneously measured in a voltage-clamped single cell, the patch pipette contained 100 μm Fura 2 pentapotassium salt, and the emitted fluorescence was captured at 510 nm using a photomultiplier (Photon Technologies International, Princeton, NJ).

##### Measurement of Flavine Adenine Dinucleotide (FAD) Fluorescence

Cultured clusters of β cells were preincubated for 60 min at 37 °C in a control medium containing 3 mm glucose and then transferred to the stage of an LSM 510 confocal microscope. After a further 10 min of perfusion by 3 mm glucose, the recording was started. The oxidized form of the FAD was excited at 488 nm. Emitted fluorescence was collected with a 505-nm long-pass filter.

##### Electrophysiology

All patch clamp measurements were carried out using a multiclamp 700B patch clamp amplifier and the software pClamp 9 (Axon Instruments, Foster City, CA). When using the perforated whole-cell mode of the patch clamp technique, the electrical contact was established by adding the pore-forming antibiotic, amphotericin B, to the pipette solution. Amphotericin (stock solution of 60 mg/ml in DMSO) was used at a final concentration of 300 μg/ml. The tip of the pipette was filled with antibiotic-free solution, and the pipette was then back-filled with amphotericin-containing solution. The voltage clamp was considered satisfactory when the access resistance was <30 megohms and stable. In the standard whole-cell configuration, the access resistance was <15 megohms. Patch pipettes were pulled from borosilicate glass capillaries (World Precision Instruments, Hertfordshire, UK); they had resistances of 3–5 megohms when filled with intracellular solution.

All experiments were carried out on single β cells. Two criteria were used to identify β cells. The capacitance of mouse α-, δ-, and β cells has been reported to be 4.4, 5, and 7.4 picofarads, respectively. Therefore, only large cells with a capacitance of >5 picofarads were chosen for this study. The average capacitance was 7.6 ± 0.2 picofarads. After verification of the capacitance, a depolarizing protocol was applied to identify the properties of the voltage-dependent Na^+^ current, which is known to be largely inactivated at resting potential in β cells but not in α and δ cells. Thus, cells in which a large Na^+^ current could be activated by a small depolarizing pulse from a holding potential of −70 mV were discarded. By contrast, cells that displayed a Na^+^ current only after a hyperpolarizing pulse to −140 mV were considered to be β cells and were used for the experiments.

The whole-cell K_ATP_ channel current (*I*_K(ATP)_) was monitored by 100-ms duration pulses of ±20 mV from a holding potential of −70 mV. Whole-cell Ca^2+^ currents were recorded by depolarizing the plasma membrane with a 100-ms pulse from −80 to 10 mV.

##### Solutions

The medium used for the isolation of islets and for all experiments was a bicarbonate-buffered solution containing (in mm) the following: 120 NaCl, 4.8 KCl, 2.5 CaCl_2_, 1.2 MgCl_2_, and 24 NaHCO_3_. It was gassed with O_2_/CO_2_ (94:6) to maintain pH 7.4 at 37 °C. Except for the electrophysiological experiments, it was supplemented with 1 mg/ml BSA (fraction V, Roche Applied Science, Mannheim, Germany). When the concentration of KCl was increased, the concentration of NaCl was correspondingly decreased to keep the osmolarity of the medium unchanged.

For electrophysiological measurements of *I*_K (ATP)_, the standard extracellular solution contained (in mm) the following: 140 NaCl, 4.8 KCl, 2.5 CaCl_2_, 1.2 MgCl_2_, 5 HEPES (pH adjusted to 7.40 with NaOH), and 10 mm glucose. These solutions were gassed with O_2_/CO_2_ (94:6%). For the perforated patch measurements of membrane currents and potential, the pipette solution contained (in mm) the following: 70 K_2_SO_4_, 10 NaCl, 10 KCl, 3.7 MgCl_2_, and 5 HEPES (pH adjusted to 7.1 with KOH). For whole Ca^2+^ current, the pipette solution consisted of (in mm) the following: Cs_2_SO_4_ substituted for K_2_SO_4_. For NAADP infusion experiments, the pipette solution contained (in mm) the following: 125 K^+^ gluconate, 10 KCl, 10 NaCl, 10 KCl, 1 MgCl_2_, 3 Mg-ATP, 0.1 Na-GTP, and 5 HEPES (pH adjusted to 7.1 with KOH). In Fig. 3*A*, 100 μm Fura 2 pentapotassium was added.

##### Gene Expression Analysis

Total RNA was extracted from mice pancreas and liver following the RNeasy QiaRNA extraction procedure, including a DNase treatment (Qiagen). RT-PCR was performed in a reaction containing extracted RNA, the SuperScriptII One-Step RT-PCR system with Platinum Taq (Invitrogen), and the following gene-specific primers: *Tpcn2* exons 4–8 amplicon (forward, 5′-gggcttcatcattttcctga-3′; reverse, 5′-ttgttggaagtcgtcagcag-3′). The following parameters were used for RT: 50 °C (30 min), 94 °C (2 min); PCR, 30 cycles of 94 °C (15 s), 57 °C (30 s), and 68 °C (1 min), followed by a final extension at 68 °C (10 min). For gene expression analysis in β cells, cDNA was produced from total RNA using the High Capacity cDNA reverse transcription kit (Applied Biosystems), and PCR was performed with the following gene-specific primers for *Tpcn2* exons 22–25 amplicon (forward, 5′-aacgtgatggtggtgaacaat-3′; reverse, 5′-gtctgccaaagctacaccttg-3). The following parameters were used for PCR: 30 cycles of 95 °C (30 s), 53 °C (30 s), and 72 °C (1 min), followed by a final extension at 72 °C (10 min). *Tpcn1* mRNA expression was analyzed as described previously (52).

##### Insulin Secretion

Islets were isolated from mice and cultured in RPMI 1640 medium overnight before insulin secretion was assessed. Insulin secretion was measured during 1-h static incubations in Krebs-Ringer Buffer (KRB) containing (in mm) the following: 18.5 NaCl, 2.54 CaCl_2_, 1.19 KH_2_PO_4_, 4.74 KCl, 25 NaHCO_3_, 1.19 MgSO_4_, and 10 HEPES (pH 7.4). Samples of the supernatant were assayed for insulin using a mouse insulin ELISA kit (Mercodia, Sweden). Where Ned-19 was used, islets were preincubated for 5 min with the drug prior to the addition of secretagogues.

##### Glucose Tolerance Tests

Male *Tpcn1*^−/−^ (*Tpcn1*^tm1Dgen^) (52) and *Tpcn2*^−/−^ (*Tpcn2*^Gt(YHD437)Byg^) (29) mice and strain-matched wild types aged 66–76 days were fasted overnight and then given 2 g/kg intraperitoneal glucose (in the form of an autoclaved 20% glucose solution). Blood samples taken from the tail vein at 0, 15, 30, 60, and 120 min were analyzed with an Accu-Chek Compact Plus glucose monitor. Mean values for each time were compared using the Student's *t* test, with *p* < 0.05 taken as significant.

##### Insulin Secretion from Whole Pancreata

Pancreatic perfusions were performed within 15 min of cervical dislocation in 80–100-day-old mice essentially as described elsewhere (53). At the end of the perfusion, the pancreas was dissected and transferred in acid/ethanol (ethanol/H_2_O/HCl, 52:17:1). All samples were then stored at −20 °C. Only experiments with an output rate greater than 200 ml/min were assayed for insulin. The hormone assay was done using a commercially available RIA kit (Millipore, Watford, UK).

##### TPC2 Localization Studies

Human islets were prepared from beating heart donors with appropriate ethical permission and consents as described previously (54). Islets were dissociated into single cells and, after fixation in 4% (w/v) paraformaldehyde, were treated with antibodies as below (55). Rabbit anti-TPC2 antibody (1:150) was revealed with Alexa 568-conjugated secondary antibody (1:1500, Invitrogen, Paisley, UK). Guinea pig anti-insulin (1:300, DAKO, Ely, UK), goat anti-EEA1 (1:150, Santa Cruz Biotechnology, Santa Cruz, CA), and rat anti-LAMP-1 (1:150, Santa Cruz Biotechnology) were revealed with Alexa 488 secondary antibodies (1:1500, Invitrogen). Murine MIN6 clonal β cells (56) were transfected with plasmid encoding TPC2-mCherry using Lipofectamine 2000, and 48 h later were fixed, stained, and imaged as above. Images were captured using a Zeiss Axiovert 200 M spinning disc confocal imaging system (×40 oil immersion objective corrected for chromatic aberration; Hamamatsu ImageEM 9100-13 back-illuminated EM-CCD camera) with illumination (491 and 568 nm) provided by solid state lasers (Crystal Laser, NV) using a laser merge module (Spectral Applied Physics, Ontario, Canada) (57).

##### Electron Microscopy

The islet preparations were fixed in 2% paraformaldehyde and 2% glutaraldehyde in cacodylate buffer in the presence of calcium chloride, washed, post-fixed in 1% osmium tetroxide, and contrast-enhanced by staining *en bloc* with uranyl acetate. Sections were further contrasted with Reynold's lead.

##### Chemicals

Ned-19, Ned-20 (21), and NAADP-AM (51) were synthesized in-house as described previously. Bafilomycin was from LC Laboratories, and other chemicals were from Sigma.

## Results

### 

#### 

##### Characterization of NAADP-AM-evoked Ca^2+^ Release

**FIGURE 1.**
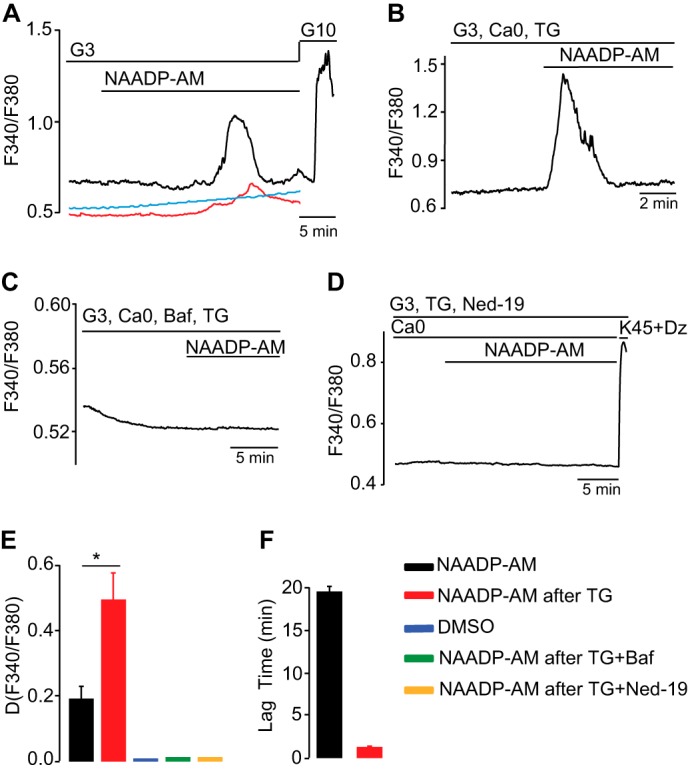
**NAADP mobilizes Ca^2+^ from acidic Ca^2+^ stores in mouse primary β cells.** *A,* clusters of β cells were superfused with 3 mm glucose and stimulated by NAADP-AM, 10 nm (*red line*), 60 nm (*black line*), and 10 μm (*blue line*), and glucose (10 mm) as indicated. *B,* clusters of β cells were pre-treated with 1 μm thapsigargin (*TG*) for 1 h. They were then stimulated by NAADP-AM (60 nm) in the absence of extracellular Ca^2+^ and in the presence of a nonstimulatory glucose concentration (3 mm). *C,* NAADP-AM-induced [Ca^2+^]*_i_* response observed in *B* is prevented by bafilomycin (*Baf*, 3 μm) treatment. *D,* pretreatment of β cells with Ned-19 (100 μm) blocks the NAADP-AM-induced [Ca^2+^]*_i_* rise observed in *B. E* and *F,* quantification of the results from *A* to *D. Traces* are representative of results obtained in 8 (*A*), 7 (*B*), 6 (*C*), and 6 (*D*) clusters of islet β cells.

**FIGURE 2.**
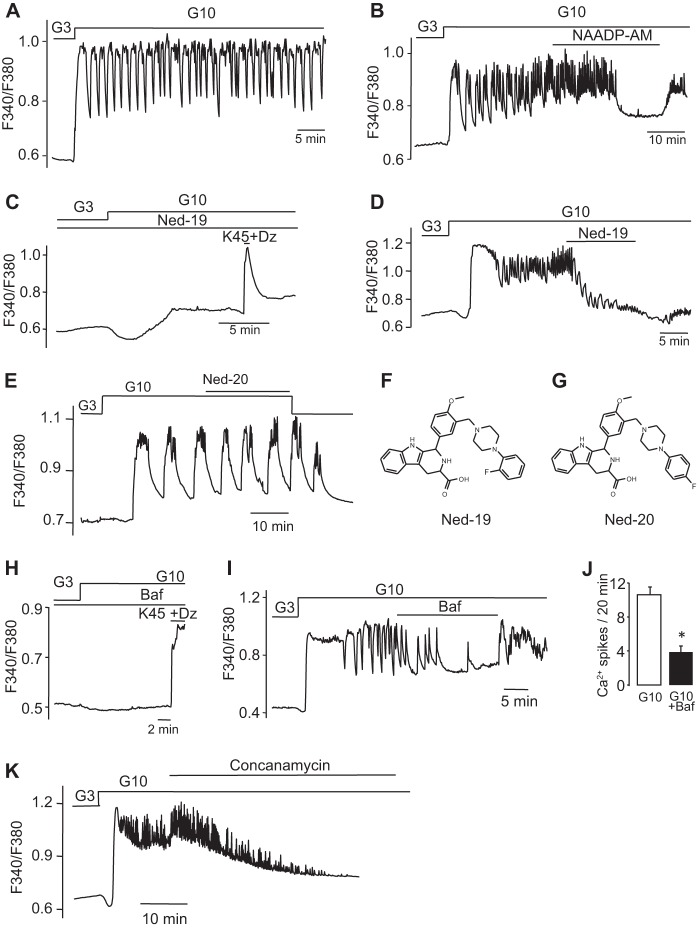
**Glucose-induced [Ca^2+^]*_i_* response in pancreatic β cells are dependent on NAADP-evoked Ca^2+^ release from acidic stores.** *A,* typical [Ca^2+^]*_i_* oscillations induced by the stimulation of clusters of islet β cells by an increase of glucose from 3 to 10 mm as indicated. *B,* clusters of β cells were challenged with 10 mm glucose, and NAADP-AM (60 nm) was added acutely as indicated. *C,* clusters of islet β cells were challenged with 10 mm glucose in the continuous presence of Ned-19. High K^+^ was applied as indicated. *D,* clusters of β cells were stimulated by an increase of glucose from 3 to 10 mm glucose, and Ned-19 was applied acutely as indicated. *E,* clusters of β cells were stimulated by an increase of glucose from 3 to 10 mm, and Ned-20 was added as indicated. Representative trace was obtained in five separate clusters of islet β cells. *F* and *G,* structures of Ned-19 and Ned-20, a close structural analogue in which the fluorine atom is para on the benzene ring. *H,* clusters of islet β cells were pretreated with 3 μm bafilomycin and challenged with glucose or K^+^ (45 mm) as indicated by *horizontal bars* (by opening the K_ATP_ channels, diazoxide (*Dz*) (100 μm) prevents the direct effect of glucose on the membrane potential). *I,* glucose-induced [Ca^2+^]*_i_* oscillations are reversibly abolished by acute addition of 3 μm bafilomycin (*Baf*). *J,* quantification of the frequency of [Ca^2+^]*_i_* oscillations in *I. K,* clusters of pancreatic β cells were challenged with 10 mm glucose, and concanamycin (6 μm) was added acutely as indicated by the *horizontal bar*. The *trace* is representative of results obtained from eight clusters of islet β cells. Other *traces* are representative of results obtained in 11 (*A*), 5 (*B*), 8 (*C*), 9 (*D*), 8 (*H*), and 10 (*I*) clusters of islet β cells (*, *p* < 0.05, Student's *t* test).

We first investigated the effects of NAADP on intracellular Ca^2+^ concentrations ([Ca^2+^]*_i_*) in primary mouse pancreatic β cells using the membrane-permeant NAADP analogue NAADP-AM (51). Given that the concentration-response curve is bell-shaped in mammalian cells (58), different NAADP-AM concentrations were tested to optimize the response (Fig. 1*A*) (58). 10 nm NAADP-AM gave only a small response, whereas 10 μm NAADP-AM gave no response at all. An intermediate concentration of 60 nm was found to give the most consistent and largest Ca^2+^ response and was used for subsequent studies. In 8/10 clusters of β cells, extracellular application of NAADP-AM (60 nm), in the presence of low glucose (3 mm), evoked delayed [Ca^2+^]*_i_* increases (Fig. 1*A*). The peak was reached >15 min after application of NAADP-AM (Fig. 1*F*). To assess the role of the ER in the action of NAADP-AM (59), we treated the cells with the SERCA pump inhibitor thapsigargin in the absence of extracellular Ca^2+^ to remove functional ER Ca^2+^ stores. In keeping with earlier observations (13), the NAADP-AM-evoked [Ca^2+^]*_i_* transients were larger and occurred more rapidly after thapsigargin treatment (Fig. 1, *B*, *E,* and *F*) (13). Previous studies have implicated acidic Ca^2+^ stores as the principal target organelles for NAADP in pancreatic β cells and other cells (19, 60). Accordingly, bafilomycin (which inhibits Ca^2+^ uptake into acidic stores dependent on V-H^+^-ATPase activity (60)) blocked Ca^2+^ release in response to NAADP-AM treatment (Fig. 1, *C* and *E*). In addition, the membrane-permeant NAADP antagonist Ned-19 (100 μm) (Fig. 2*F*) (21) also completely abolished the Ca^2+^ transient evoked by NAADP-AM (Fig. 1, *D* and *E*). Collectively, these data suggest that NAADP targets acidic Ca^2+^ stores rather than the ER in mouse pancreatic β cells.

##### Modulation of Glucose-evoked Ca^2+^ Spiking by Ned-19 and Vacuolar Proton Pump Inhibitors

We next examined whether NAADP signaling plays a role in glucose-mediated responses in primary β cells as suggested previously in MIN6 cells (18). Stimulation of mouse pancreatic β cells by 10 mm glucose resulted in [Ca^2+^]*_i_* oscillations that were superimposed upon a sustained plateau (Fig. 2*A*). Acute application of 60 nm NAADP-AM first enhanced Ca^2+^ spiking from 5.8 ± 0.7 to 11.6 ± 1.3 spikes/min (*n* = 5; *p* < 0.01) and then abolished glucose-evoked [Ca^2+^]*_i_* oscillations after about 20–25 min (Fig. 2*B*). This finding is consistent with the bell-shaped concentration-response curve to NAADP in mammalian cells (18, 51, 61); the initial stimulatory effect was mediated by low concentrations, and the subsequent inhibition reflected the build-up of higher self-desensitizing concentrations of NAADP (16, 18). The antagonist (18, 51, 61) Ned-19 was also found to inhibit the glucose-induced [Ca^2+^]*_i_* rise, and it abolished the Ca^2+^ oscillations, without affecting the initial [Ca^2+^]*_i_* decrease due to ATP-enhanced Ca^2+^ uptake by the ER (Fig. 2*C*) (62). When Ned-19 was applied after commencement of glucose-evoked Ca^2+^ responses, the glucose-induced [Ca^2+^]*_i_* plateau was abolished (Fig. 2*D*). The structurally related analogue Ned-20, which is not an NAADP antagonist (Fig. 2*G*) (21), was without effect (Fig. 2*E*).

Because NAADP mobilized Ca^2+^ from acidic organelles (Fig. 1*C*) (60), we examined whether selective pharmacological interference of Ca^2+^ storage by acidic organelles with agents that affect Ca^2+^ uptake into these stores (60) modulates glucose-evoked Ca^2+^ signaling. Preincubation of β cells with bafilomycin prevented the glucose-induced [Ca^2+^]*_i_* rise (Fig. 2*H*), as observed previously in the MIN6 β cell line (19). When bafilomycin was applied acutely, it reduced glucose-induced [Ca^2+^]*_i_* oscillations (Fig. 2*I*). Similar results were obtained with concanamycin, another V-type-H^+^-ATPase blocker (Fig. 2*K*). The effect of bafilomycin on the frequency of glucose-evoked [Ca^2+^]*_i_* transients is summarized in Fig. 2*J*. These observations are consistent with the hypothesis that NAADP-sensitive acidic Ca^2+^ stores play a key role in sustaining glucose-induced [Ca^2+^]*_i_* oscillations in mouse pancreatic β cells (63).

##### NAADP Modulation of Plasma Membrane Currents and Membrane Potential

**FIGURE 3.**
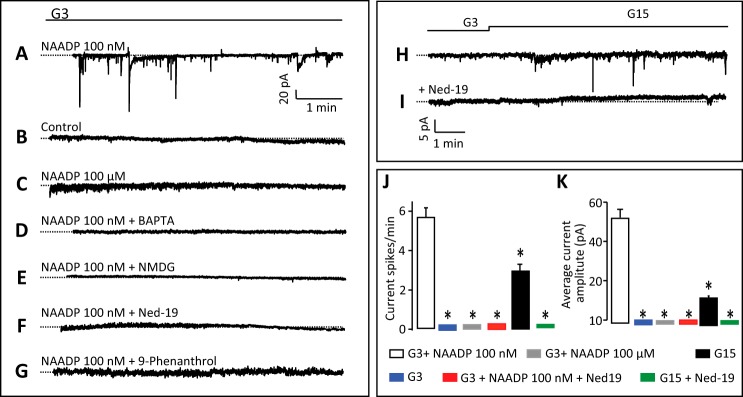
**NAADP evokes Ca^2+^-dependent inward currents in pancreatic β cells.** *A,* inward currents at −70 mV evoked by the infusion of 100 nm NAADP through a patch pipette in the standard whole-cell configuration. *B–G,* currents are absent under control conditions (*i.e.* in the absence of NAADP in the pipette solution (*B*); in the presence of 100 μm NAADP in the pipette solution (*C*); in the presence of 10 mm Ca^2+^ chelator BAPTA alongside 100 nm NAADP in the patch pipette (*D*); when the positive ions in the extracellular solution were replaced with *N*-methyl-d-glucamine (*E*); in the presence of extracellular Ned-19 (100 μm) (*F*); and the presence of the TRPM4 channel inhibitor 9-phenanthrol (10 μm) (*G*)). The *dotted lines* represent the zero current level. *H* and *I,* glucose-induced inward currents in single cells clamped at −70 mV in the absence (*H*) or presence (*I*) of Ned-19 (100 μm). The *dotted lines* represent the zero current level. *J* and *K,* quantification of data from *A* to *I* and showing frequencies (*J*) and amplitudes (*K*) of currents. *Traces/histograms* are representative of or were obtained from 10 (*A*), 18 (*B*), 11 (*C*), 8 (*D*), 9 (*E*), 11 (*F*), 5 (*G*), 4 (*H*), 4 (*I*). *, *p* < 0.05.

We have previously shown that intracellular application of NAADP in β cells evokes oscillatory currents (21, 29). To examine the impact of NAADP-evoked Ca^2+^ release on the excitability of pancreatic β cells in more detail, we infused NAADP (10–100 nm) through a patch pipette in the standard whole-cell configuration while simultaneously measuring the membrane currents. In β cells clamped at −70 mV, intracellular application of 100 nm NAADP evoked intermittent inward currents of varying amplitudes (Fig. 3*A*). These currents contrast with the periodic outward hyperpolarizing K^+^ currents evoked by IP_3_-evoked Ca^2+^ release in β cells (64), and they underscore the differential actions of these two Ca^2+^-mobilizing messengers. The inward currents were absent in control cells where no NAADP was added to the intracellular solution (Fig. 3*B*), as well as when highly desensitizing concentrations (>1 μm) of NAADP were used (Fig. 3*C*), consistent with the bell-shaped concentration-response curve due to self-desensitization of the NAADP receptor (18, 61). Moreover, these currents were also prevented by addition of the Ca^2+^ chelator BAPTA to the pipette solution, demonstrating that they are likely dependent on Ca^2+^ release (Fig. 3*D* and see Fig. 7*A*). When *N*-methyl-d-glucamine replaced extracellular K^+^ and Na^+^, no inward currents could be seen in response to 100 nm NAADP (Fig. 3*E*). Taken together with the BAPTA studies, these data are consistent with the inward currents being due to opening of Ca^2+^-activated cation channels. The NAADP antagonist Ned-19 (100 μm) also abolished the NAADP-evoked currents, as shown previously (Fig. 3*F*) (21). NAADP-evoked Ca^2+^ release has recently been reported to activate TRPM4 channels in HeLa cells (65), and because this Ca^2+^-activated nonselective cation channel has been proposed to control insulin secretion (66) along with TRPM5 (67–69), we tested the effect of 9-phenanthrol, a TRPM4 blocker (70), on the NAADP-evoked currents in β cells. We found that the currents were inhibited by this drug (Fig. 3*G*), suggesting a possible involvement of TRPM4 channels.

Importantly, like NAADP, high glucose (15 mm) concentrations also evoked spontaneous inward currents in cells voltage-clamped at −70 mV (Fig. 3*H*). These glucose-induced inward currents were also blocked by Ned-19 (100 μm) (Fig. 3*I*). The frequencies and amplitudes of the above NAADP- and glucose-evoked currents from Fig. 3, *A–I,* are summarized in Fig. 3, *J* and *K*, respectively. Taken together, these data raise the exciting possibility that NAADP-mediated Ca^2+^ release from acidic stores may modulate the glucose-mediated membrane currents that in turn initiate β cell electrical activity and insulin secretion.

**FIGURE 4.**
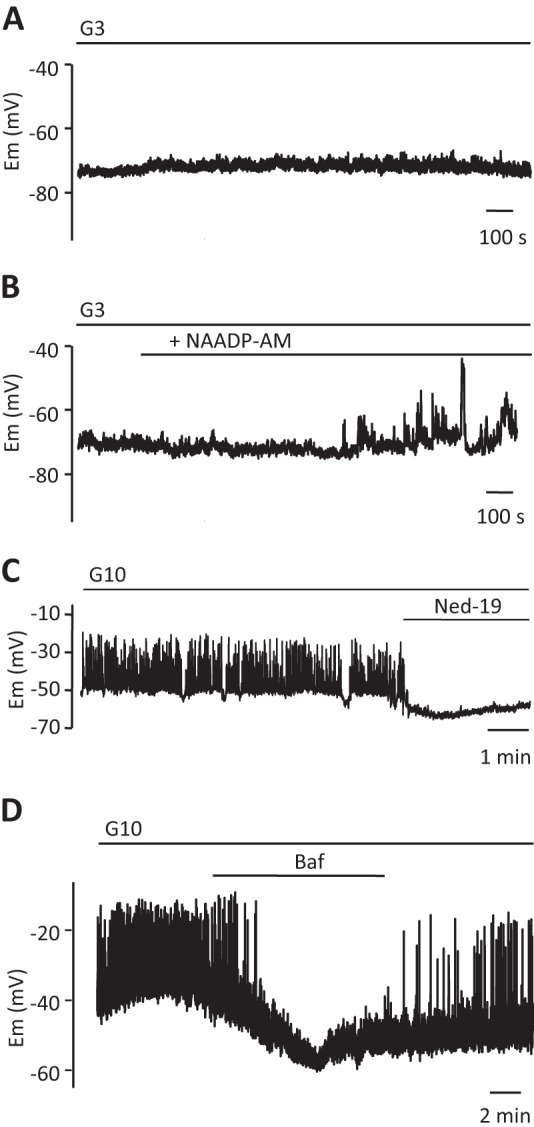
**NAADP-evoked Ca^2+^ release from acidic stores modulates membrane potential.** *A* and *B,* membrane potential (*Em*) recordings from β cells in small clusters exposed to 3 mm glucose. NAADP-AM (60 nm) was applied as indicated in *B. C,* Ned-19 (100 μm) abolishes the typical electrical activity evoked by 10 mm glucose in a single pancreatic β cell. *D,* typical electrical activity evoked by 10 mm glucose in a single β cell is reversibly abolished by bafilomycin (3 μm). *Traces* are representative of results obtained from three (*A*), six (*B*), and seven (*C* and *D*) single β cells. All *traces* represent different cells. *, *p* < 0.05, Student's *t* test.

We next examined the effect of intracellular NAADP on the pancreatic β cell plasma membrane potential. A nonstimulatory level of glucose (3 mm) alone (Fig. 4*A*) had no effect on resting membrane potential (around −70 mV). In the presence of 3 mm glucose, NAADP-AM (60 nm) evoked low amplitude voltage oscillations that did reach the threshold for action potential firing (Fig. 4*B*). At 10 mm glucose, glucose-induced electrical activity consisting of 30–40-mV action potentials was observed. Under these conditions, the blockade of NAADP action with Ned-19 (100 μm) resulted in membrane hyperpolarization and suppression of electrical activity (Fig. 4*C*).

If NAADP-evoked Ca^2+^ release is involved in the regulation of the membrane potential by glucose, pharmacological manipulation of ion fluxes across the endomembranes involved would be predicted to impact on glucose-mediated changes in membrane excitability. It is therefore of interest that bafilomycin reversibly suppressed glucose-evoked action potentials (Fig. 4*D*).

##### Lack of Effects of Ned-19 and Vacuolar Proton Pump Inhibitors on Plasma Membrane Currents and Cell Metabolism and Validation of Compound Used

Although the profound effects of Ned-19 and bafilomycin on glucose-mediated Ca^2+^ signaling and electrical changes above were ascribed to antagonism of NAADP and abrogation of acidic organelle Ca^2+^ storage, it was important to rule out other targets that could potentially account for the effects of these two agents on glucose action.

**FIGURE 5.**
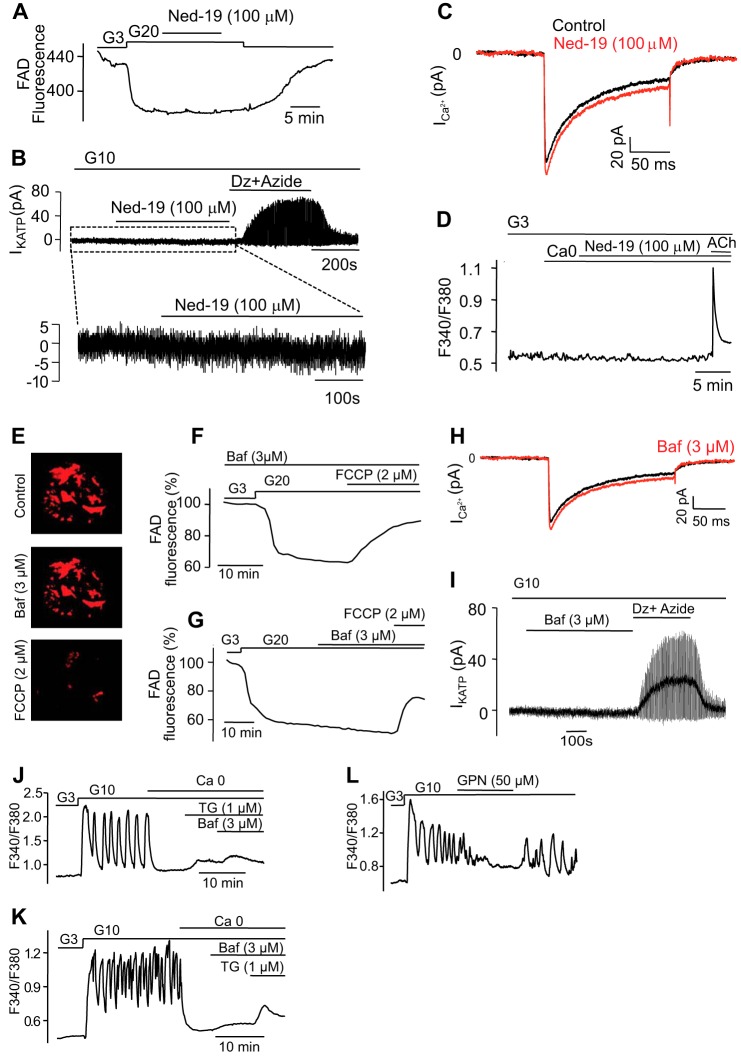
**Selectivity of pharmacological compounds employed.** Ned-19 does not affect mitochondrial metabolism, plasma membrane currents, or IP_3_-induced Ca^2+^ release. *A–C,* changes in flavin adenine dinucleotide (*FAD*) fluorescence in response to glucose, which reflect glucose metabolism (*A*), the whole-cell K_ATP_ current (*B*), and the whole-cell voltage-dependent Ca^2+^ current (*C*) produced by Ned-19 (100 μm). *D,* no effect of Ned-19 on [Ca^2+^]*_i_* increases evoked by the muscarinic agonist acetylcholine (*ACh*). *Traces* are representative of results obtained in seven (*B*) and six (*C*) single β cells or six (*A*) and six (*D*) clusters of islet β cells. Bafilomycin (*Baf*) does not impair either glucose metabolism or whole-cell Ca^2+^ or K^+^-ATP currents. *E, top,* clusters of islets β cells were loaded for 10 min with 10 nm of the potentially sensitive probe for measuring membrane potential changes in mitochondria, tetramethylrhodamine ethyl ester. An image was taken 5 min after washout of the dye. *Middle,* an image was taken after 15 min of incubation with 3 μm bafilomycin. *Bottom,* an image was taken after application of 2 μm of the protonophore, carbonyl cyanide *p-*(trifluoromethoxy) phenylhydrazone (*FCCP*), which depolarizes the mitochondrial membrane. Either pretreatment (*F*) or acute addition (*G*) of bafilomycin does not alter glucose metabolism reflected by changes in flavin adenine dinucleotide (*FAD*) fluorescence in response to glucose. Neither whole-cell Ca^2+^ current (*H*) nor whole-cell K^+^-ATP current (*I*) was affected by bafilomycin (in *H* the Ca^2+^ current was recorded from the same cell before (*black trace*) and after bafilomycin application (*red trace*)). Images in *E* are representative of results obtained in six separate experiments. *Traces* are representative of results obtained in four (*F* and *G*) clusters of β cells and seven (*H*) and five (*I*) single β cells. Bafilomycin-sensitive Ca^2+^ stores are separate from the ER. *J* and *K,* clusters of islet β cells were transferred to a Ca^2+^-free solution before 1 μm thapsigargin (*Tg*), and 3 μm bafilomycin was added as indicated by *horizontal bars*. Representative *traces* of results obtained in seven (*J*) and six (*K*) clusters of islet β cells. *L,* lysosomes are essential for glucose-induced [Ca^2+^]*_i_* oscillations. Clusters of islet β cells were stimulated by 10 mm glucose, and 50 μm glycyl-l-phenylalanine 2-naphthylamide (*GPN*) was applied as indicated. The GPN is a cathepsin C substrate, which permeabilizes the lysosomes by osmotic swelling. *Trace* is representative of results obtained in seven clusters of islet β cells. *Dz*, diazoxide; *ACh,* acetylcholine.

Ned-19 exerted no significant effects upon glucose metabolism, as indicated by the persistence of glucose-induced decrease in mitochondrial FAD fluorescence (Fig. 5*A*). The inhibitor was also without effect on K_ATP_ channel activity; in the presence of 10 mm glucose, the whole-cell conductance averaged 0.21 ± 0.04 and 0.20 ± 0.06 nanosiemens in the absence or presence of Ned-19 (Fig. 5*B*). By contrast, the combination of the K_ATP_ channel activator diazoxide and the mitochondrial inhibitor sodium azide (1 mm) resulted in a large increase in K_ATP_ channel activity. Similarly, there was no inhibitory effect of Ned-19 on the voltage-gated Ca^2+^ currents; the peak current during depolarization from −70 to 0 mV averaged 110 ± 1 pA (*n* = 10) and 114 ± 1 pA (*n* = 10) in the absence and presence of Ned-19, respectively (Fig. 5*C*). Thus, the suppression of electrical activity cannot simply be attributed to activation of K_ATP_ channels or inhibition of VDCCs. In addition, Ned-19 was without effect on Ca^2+^ release induced by the stimulation of muscarinic receptors with acetylcholine (100 μm) (Fig. 5*D*), which leads to the opening of IP_3_ receptors and discharge of ER stores (71). These data are therefore consistent with a high degree of selectivity of Ned-19 as an antagonist of NAADP and demonstrate that its effects are consistent with a major role for NAADP-induced Ca^2+^ release in glucose-induced Ca^2+^ signaling.

We next analyzed the effects of bafilomycin on the same functional parameters. The effects of the latter inhibitor were independent of alterations in glucose-evoked changes in mitochondrial membrane potential or cell metabolism (Fig. 5, *E–G*), VDCCs (Fig. 5*H*), or modulation of K_ATP_ channels (Fig. 5*I*). Moreover, in the absence of extracellular Ca^2+^, bafilomycin increased [Ca^2+^]*_i_* after thapsigargin treatment (Fig. 5*J*), and vice versa (Fig. 5*K*), confirming that bafilomycin-sensitive Ca^2+^ stores are distinct from the ER. The application of the lysomotropic agent GPN, a lysosomotropic agent that abrogates Ca^2+^ storage by lysosomes (19, 25), exerted effects that resembled those of bafilomycin (Fig. 5*L*), again indicating that a lysosome-related organelle is the likely source of Ca^2+^ release.

##### TPC2 Expression and Subcellular Localization in Endocrine Pancreas

**FIGURE 6.**
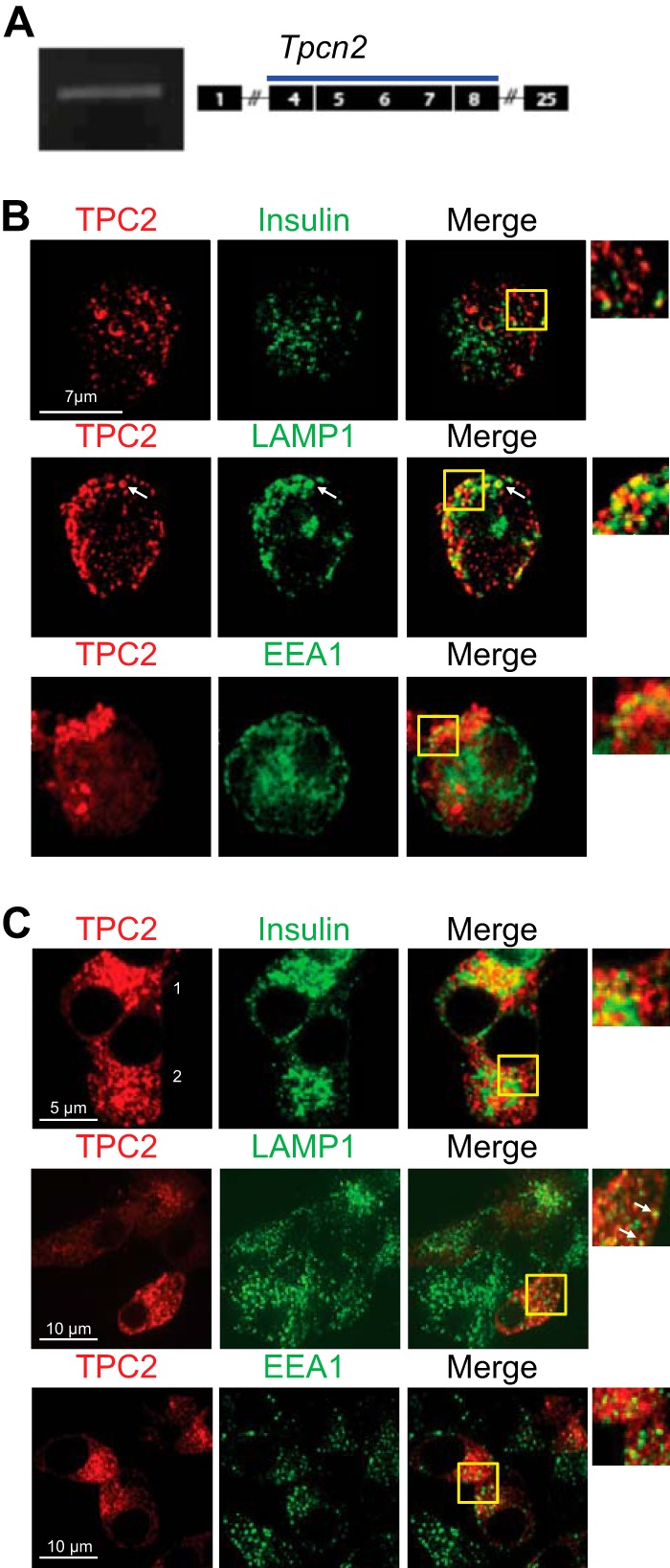
**Subcellular localization of TPC2 in human β cells and mouse MIN6 β cells.** *A,* RT-PCR product corresponding to TPC2 mRNAs in the pancreas from WT mice with expected product size of 501 bp. *B,* co-labeling of endogenous TPC2 and organelle markers in single human β cells. Single human β cells were fixed and imaged as described under “Experimental Procedures.” Zoomed images show areas within the *yellow boxes*. Although little if any co-localization was observed between TPC2 and insulin or EEA1 staining, clear overlap was apparent between TPC2 and LAMP1 in well defined subcellular structures (*arrows*). *C,* colabeling of overexpressed TPC2-mCherry and organelle markers in clonal mouse β cells. MIN6 cells were transfected with a TPC2-mCherry construct (see under “Experimental Procedures”) and subsequently fixed and permeabilized. Guinea pig anti-insulin, goat anti-EEA1, and rat anti-LAMP-1 were revealed as in *A*. Overlap between TPC2 and LAMP1-labeled structures is clearly apparent (*inset, arrows*). Limited overlap between TPC2 and insulin was observed only in cells expressing high levels of TPC2-mcherry (*cell 1*), but little if any co-labeling of insulin and TPC2 was observed in the majority of cells (*cell 2*) where TPC2-mCherry levels were lower.

Turning to the molecular targets for NAADP, we have recently identified TPC2, encoded by the *Tpcn2* gene, as a critical mediator of the NAADP response (29), and we have shown that it is required to couple stimuli to Ca^2+^ release from acidic stores (72). This channel is localized on acidic stores, but not at the ER or plasma membrane, and in particular it co-localizes with organelles of the endolysosomal system, most prominently the lysosomes (29). RT-PCR analysis of mouse islets indicates that *Tpcn2* is expressed in mouse islets (Fig. 6*A*). Affirming the localization of TPC2 to acidic stores in β cells, immunolocalization of the endogenous TPC2 in primary human β cells revealed substantial overlap with immunoreactivity of LAMP1, a major lysosomal marker (Fig. 6*B*). There was substantially less overlap with immunoreactivity for insulin or EEA1, used as markers for insulin granules (73) and endosomes (29), respectively. In a complementary approach, TPC2-mCherry was expressed in MIN6 cells (Fig. 6*C*). A similar lysosomal localization of TPC2-mCherry transfected into this murine β cell line was seen, but some co-localization with insulin granules could also be seen.

##### Glucose and Tolbutamide-evoked Ca^2+^ Responses Are Reduced in Isolated Pancreatic β Cells from Tpcn2^−/−^ Mice

**FIGURE 7.**
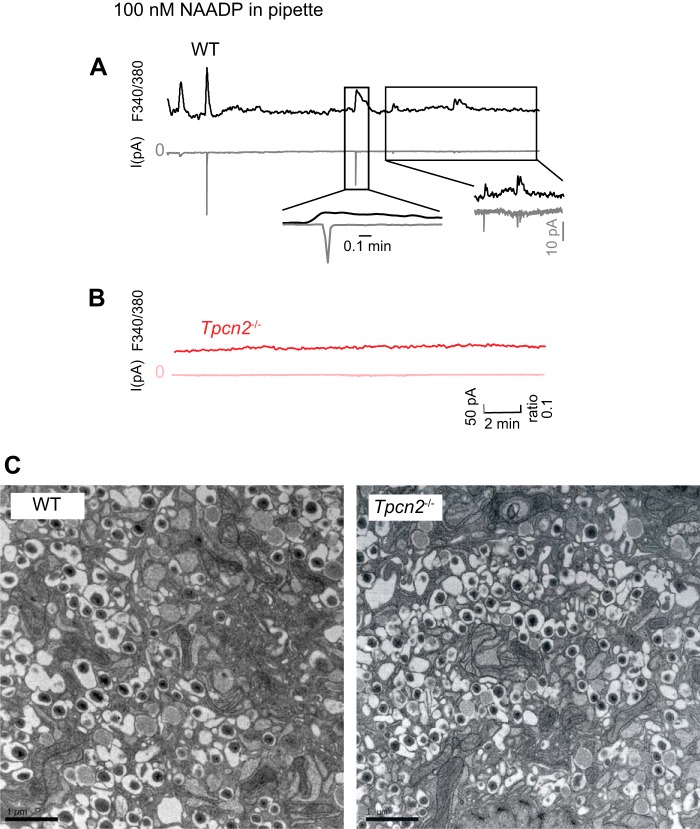
**NAADP evokes membrane currents and Ca^2+^ signals in pancreatic β cells from wild-type but not *Tpc2*^−/−^ mice.** *A* and *B,* simultaneous [Ca^2+^]*_i_* (*black trace, upper*) and whole-cell current recording (*gray trace, lower*) in response to the infusion of 100 nm NAADP through a patch pipette from a wild type (*A*) or *Tpcn2*^−/−^ (*B*) single β cells voltage clamped at −70 mV. *Traces* are representative of results obtained from four (*A*) and four (*B*) single β cells. *C,* electron micrographs of mouse pancreatic β cells from wild-type and *Tpcn2*^−/−^ mice. EM sections are shown of β cells from pancreatic islet preparations from *Tpcn2*^−/−^ knock-out mice as indicated. *Scale bar,* 1 μm.

We next examined the effects of infusing NAADP (100 nm) into mouse pancreatic β cells from age-, sex-, and background-matched wild-type and *Tpcn2*^−/−^ mice via the patch pipette during standard whole-cell recordings while simultaneously measuring intracellular Ca^2+^ concentrations (by Fura 2) and inward membrane currents. In wild-type β cells, NAADP (100 nm) evoked [Ca^2+^]*_i_* transients, some of which associated with transient inward currents (Fig. 7*A*). However, both NAADP-evoked Ca^2+^ transients and currents were absent in cells prepared from *Tpcn2*^−/−^ mice (Fig. 7*B*), mirroring the effect of Ned-19 on the wild-type cells (Fig. 3*F*). Detailed morphological comparison of β cells from *Tpcn2*^−/−^ and wild-type mice by electron microscopy indicated no substantial differences in morphology, organelle number, or distribution, including those of insulin granules (Fig. 7*C*), making it unlikely that changes in these parameters were the underlying cause for alterations in NAADP responses observed in *Tpcn2*^−/−^ null β cells.

**FIGURE 8.**
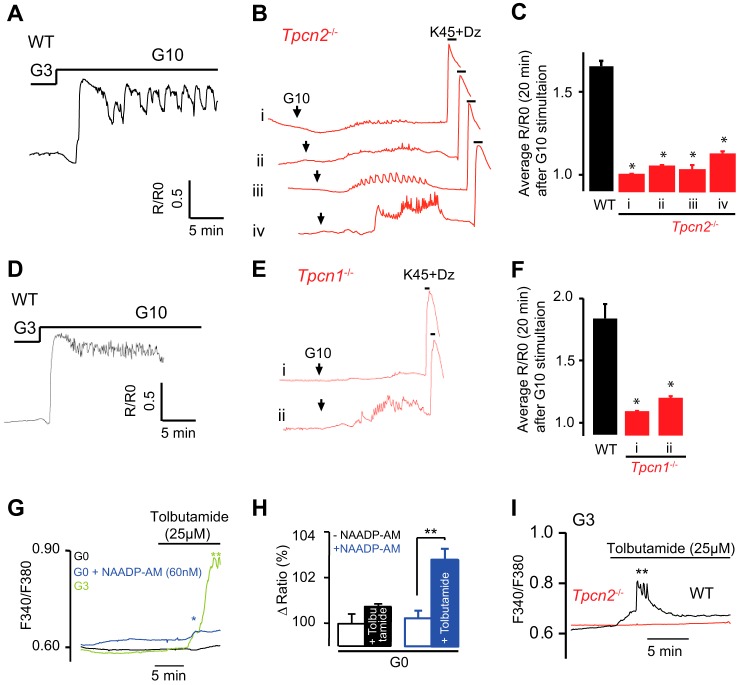
**Glucose-evoked Ca^2+^ signals are impaired in pancreatic β cells from *Tpcn2*^−/−^ and *Tpcn1*^−/−^ mice.** *A* and *B,* [Ca^2+^]*_i_* oscillations in response elevating glucose from a basal 3 mm to 10 mm glucose in wild-type (*A*) and *Tpcn2*^−/−^ β cells (*B*). *B,* experiments were concluded by addition of high extracellular K^+^ (45 mm) in combination with diazoxide (*Dz*) (100 μm). *C,* averaged *R*/*R*_0_ changes in WT and *Tpcn2*^−/−^ clusters of β cells (four different patterns were observed in *Tpcn2*^−/−^ cells) over a period of 20 min after the rise of glucose concentration from 3 to 10 mm. The changes in [Ca^2+^]*_i_* are displayed as the normalized ratio *R*/*R_0_* (*R*_0_ is the basal level before the stimulation with high glucose). *Traces* are representative of results obtained from four (*A*) and four (*B*) single β cells and 23 (*A*) and 29 (*B*) clusters of β cells. (*, *p* < 0.05, Student's *t* test.) *D–F,* averaged *R*/*R*_0_ changes in clusters of β cells from WT and *Tpcn1*^−/−^ mice (two different patterns were observed in *Tpcn1*^−/−^ cells) over a period of 20 min after the rise of glucose concentration from 3 to 10 mm. *Traces* are representative of results obtained from 11 (*D*) and 13 (*E*) clusters of β cells. (*, *p* < 0.05, Student's *t* test.) *G,* clusters of β cells were bathed in different conditions as indicated by different colors, and tolbutamide (25 μm) was applied as indicated. The sulfonylurea was unable to evoke a [Ca^2+^]*_i_* rise in the absence of glucose (*black trace, n* = 5), although a significant [Ca^2+^]*_i_* rise is observed in the presence of 3 mm glucose (*green trace, n* = 7). 30 min pretreatment with low concentrations of NAADP-AM (60 nm) (*blue line, n* = 6) permitted a [Ca^2+^]*_i_* response to tolbutamide in the absence of glucose. *H,* NAADP-AM permits 25 μm tolbutamide to rise [Ca^2+^]*_i_* in the absence of glucose. The change in ratio is expressed in %, the baseline before addition of tolbutamide being 100%. **, *p* < 0.01. *I,* clusters of pancreatic β cells were isolated from wild-type (*black trace,* representative of *n* = 5) and *Tpcn2*^−/−^ mice (*red trace,* representative of *n* = 7) and bathed in media containing 3 mm glucose. Tolbutamide (25 μm) was added as indicated by the *horizontal bar*. (*, *p* < 0.05, Student's *t* test.)

Next, we examined the Ca^2+^ responses to glucose of β cells prepared from wild-type and *Tpcn2*^−/−^ mice (Fig. 8, *A* and *B*). In *Tpcn2*^−/−^ β cells, glucose-evoked Ca^2+^ transients were either abolished, reduced in amplitude, or delayed (Fig. 8, *B* and *C*) compared with the robust responses observed in wild-type cells (Fig. 8, *A* and *C*). The average [Ca^2+^]*_i_* rises evoked by high glucose were substantially reduced (but *not* abolished) in all *Tpcn2*^−/−^ β cells studied (Fig. 8*C*), although activation of VDCCs by membrane depolarization by K^+^ (45 mm) in the presence of the K_ATP_ channel opener, diazoxide (100 μm), still evoked a large [Ca^2+^]*_i_* response (Fig. 8*B*). Similar results were obtained from *Tpcn1*^−/−^ β cells (Fig. 8, *D–F*). These studies indicate that the lysosomal TPC2 and endosomal TPC1 channels play a significant role in the generation of glucose-induced Ca^2+^ signals in pancreatic β cells, likely through their modulation of β cell electrical activity.

It has been noted that a minimum concentration of glucose “fuel” is required for threshold concentrations of the oral hypoglycemic agent and K_ATP_ inhibitor tolbutamide to mimic the electrical effects of raised glucose levels in mouse β cells (74). In agreement, we also found that in the absence of glucose (0 mm), tolbutamide (25 μm) treatment failed to increase [Ca^2+^]*_i_*, in contrast to the effect in the presence of 3 mm glucose. However, pretreatment with NAADP-AM (60 nm) partially reconstitutes the Ca^2+^ signal with tolbutamide in the absence of glucose (Fig. 8, *G* and *H*). Furthermore, we found that in wild-type mouse β cells exposed to 3 mm glucose, tolbutamide (25 μm) evoked a rise in [Ca^2+^]*_i_* (Fig. 8*I*), whereas cells from *Tpcn2*^−/−^ mice failed to respond to tolbutamide.

##### Role of NAADP and TPC2 in Glucose-induced Insulin Secretion

**FIGURE 9.**
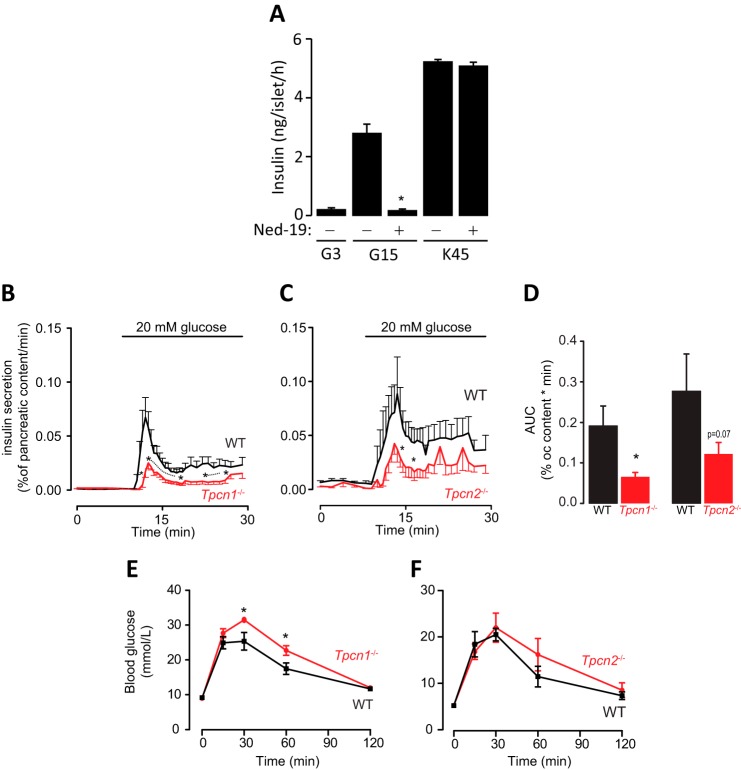
**Role of the NAADP-sensitive Ca^2+^ stores and the two-pore channel 2 (TPC2) in stimulus-secretion coupling in pancreatic β cells.** *A,* Ned-19 blocks glucose-evoked insulin secretion. Insulin secretion from control intact islets of Langerhans was triggered by glucose (15 mm) or K^+^ (45 mm). When Ned-19 was used, the islets were pretreated for 5 min prior to stimulation with the secretagogues. Data are means ± S.E. obtained from three different islet preparations with batches of 10 islets. * indicates *p* < 0.05 preparations with batches of 10 islets. *B,* insulin secretion from perfused pancreata from WT and *Tpcn1*^−/−^ mice in response to a rise of glucose from 3 to 20 mm at 8 min. The secretion is expressed as percentage of pancreatic content/min. Wild-type trace (*WT*) is shown in *black* and knock-out (*KO*) animals in *red. C,* insulin secretion from perfused pancreata from WT and *Tpcn2*^−/−^ mice in response to a rise of glucose from 3 to 20 mm at 8 min. The secretion is expressed as percentage of pancreatic content/min. Wild-type trace (*WT*) is shown in *black* and knock-out (*KO*) animals in *red. D,* area under the curve (*AUC*) for 1st and 2nd phase insulin secretion. The *traces* show the average ± S.E. of *n* = 6 WT and *n* = 6 for *Tpcn1*^−/−^ and *Tpcn2*^−/−^. * indicates a *p* < 0.07 significance level between WT and KO using a one-sided *t* test. *E* and *F,* glucose tolerance tests. Glucose tolerance test was performed on WT (*n* = 5) and *Tpcn1*^−/−^ and *Tpcn2*^−/−^ (*n* = 6) animals by intraperitoneal injection of 2 g/kg glucose solution after an overnight fasting. Results are expressed as concentrations of blood glucose at time 0 for each animal. Values are means ± S.E. of results obtained with mice for each group.

Having implicated a key role for NAADP, TPC2, and lysosomal Ca^2+^ stores in Ca^2+^ signaling and electrical activity, we finally examined the effect of disrupting NAADP signaling upon insulin secretion itself. Insulin secretion was measured from isolated whole islets in response to glucose or 45 mm K^+^. Prior treatment of islets with Ned-19 (100 μm) for 5 min substantially inhibited insulin secretion induced by 15 mm glucose but not by 45 mm K^+^ (Fig. 9*A*). The absence of an effect of Ned-19 on insulin secretion induced by high K^+^ makes it unlikely that there is a relevant off-target effect of the inhibitor upon the exocytotic machinery.

We then studied insulin secretion evoked by 20 mm glucose using the perfused pancreas preparation in wild-type, *Tpcn1*^−/−^, and *Tpcn2*^−/−^ mice. In the *Tpcn1*^−/−^ and *Tpcn2*^−/−^ pancreata, both the 1st (the response during the initial 7 min) and 2nd phase release (insulin secretion once the 1st phase release had ended) were reduced by ∼50% (Fig. 9, *B–D*).

Finally, we performed intraperitoneal blood glucose tests in fasted TPC knock-out mice in comparison with the corresponding strain-matched wild-type animals to examine the effect of perturbing TPC expression upon glucose homeostasis in the whole animal. This revealed that in *Tpcn1*^−/−^ mice (Fig. 9*E*) the blood glucose levels peaked at significantly higher levels than WT, and the time course revealed an impaired glucose tolerance capacity. In contrast, *Tpcn2*^−/−^ mice (Fig. 9*F*) were less affected.

## Discussion

This study highlights the importance of NAADP-sensitive acidic stores and the newly identified endolysosomal channels TPC1 and TPC2 in Ca^2+^ signaling during stimulus-secretion coupling in mouse pancreatic β cells. Since its discovery as a potent Ca^2+^-mobilizing agent in sea urchin egg homogenates (75), NAADP has been widely demonstrated to evoke Ca^2+^ signals in an extensive range of mammalian cells, including those of both the endocrine and exocrine pancreas (76). NAADP is an alternative product of multifunctional ADP-ribosyl cyclase enzymes, which is also responsible for the synthesis of the ryanodine receptor-regulating messenger cADPR (46). Building on early studies suggesting that cADPR is an important regulator of Ca^2+^ signaling during secretion-coupling in pancreatic β cells (77), we now reported that NAADP also mobilizes Ca^2+^ in pancreatic β cells (16–18).

In contrast to the other two principal mobilizing messengers IP_3_ and cADPR, the major target organelles for NAADP in sea urchin eggs are acidic stores rather than the ER. Pharmacological approaches and cell fractionation studies revealed that NAADP releases Ca^2+^ from a separate organelle to the ER (78, 79), identified as acidic lysosomally related organelles (25). This principle was later extended to mammalian cells and NAADP release from acidic stores and has now been established in a large number cell types (60, 80). In many cells, signaling domains at lysosome-ER junctions have been observed (81). Thus, NAADP-evoked Ca^2+^ release from acidic stores may trigger further Ca^2+^ release from larger ER Ca^2+^ stores through the mediation of IP_3_ receptors and ryanodine receptors (59). Here, we have found that Ca^2+^ signals evoked by the membrane-permeant NAADP analogue NAADP-AM are from intracellular stores because they persist in the absence of extracellular Ca^2+^ and that the NAADP antagonist Ned-19 blocks this effect (Fig. 1).

Comparison of the effects of drugs that effect Ca^2+^ uptake and storage in different organelles supports a role for acidic stores rather than the ER as the target of NAADP. Bafilomycin selectively inhibits vacuolar H^+^ pumps that acidify acidic stores, and it has been shown that Ca^2+^ uptake into acidic organelles is pH-dependent and probably mediated by Ca^2+^/H^+^ exchange (60). Bafilomycin treatment was thus found to abolish NAADP-AM-evoked Ca^2+^ release (Fig. 1*C*). In contrast, thapsigargin (a SERCA pump inhibitor that blocks Ca^2+^ uptake into the ER) was found to enhance NAADP-AM-induced Ca^2+^ release. This suggests that NAADP-evoked Ca^2+^ release in the β cell does not trigger further Ca^2+^ release through ER mechanisms. Rather the predominant role of the ER here is to act to buffer Ca^2+^ rather than as a source for release, and the functional removal of the ER decreases Ca^2+^ buffering, allowing Ca^2+^ release from acidic stores to increase further in the cytoplasm. The role of the ER to buffer Ca^2+^ during signaling has also been noted for glucose-evoked Ca^2+^ signals where glucose first decreases cytoplasmic Ca^2+^ due to increased ATP generation and stimulation of SERCA pumps (12, 82, 83). Previous studies also support acidic stores as targets for NAADP. Ca^2+^ indicators targeted to acidic granules or ER in MIN6 cells showed that NAADP releases Ca^2+^ from acidic organelles but not the ER (17). Bafilomycin and the lysosomotropic agent GPN abolishes Ca^2+^ release by photolysis of caged NAADP in MIN6 cells, but it does not affect IP_3_-evoked Ca^2+^ release (19). In primary mouse β cells, NAADP-evoked Ca^2+^ release was inhibited by GPN, which was shown to lyse acidic stores selectively (13, 23). The delay in Ca^2+^ responses seen with NAADP-AM (Figs. 1 and 4) may also be determined partly by the time for hydrolysis of ester groups by intracellular endogenous esterases, which varies between cells (51), but the delay is probably largely due to initial buffering of Ca^2+^ by the ER because it was decreased by thapsigargin treatment. In addition, by analogy with the situation in the ER (83), uptake of Ca^2+^ into acidic stores might be enhanced by glucose-stimulated ATP production, and because luminal Ca^2+^ sensitizes TPCs to low NAADP concentrations, this could promote Ca^2+^ release from these stores (84, 85), an effect that would be enhanced by removing competing ER stores.

Since the initial reports linking NAADP-evoked Ca^2+^ release to two-pore channels (29–31), there have been numerous reports of TPCs playing an essential role in mediating NAADP-evoked Ca^2+^ release from acidic stores (58, 86). However, recent evidence points to a separate NAADP-binding protein that interacts with TPCs to confer NAADP sensitivity (39). Indeed, the requirement of NAADP-binding proteins may suggest that under certain circumstances these proteins may interact with multiple channel types (87). This may explain a recent report of the loss of sensitivity of isolated lysosomes to NAADP (23, 38), and the finding that glucose may apparently still evoke Ca^2+^ signals in β cells from *Tpcn1*^−/−^*/Tpcn2*^−/−^ mice (23), although it should be noted that the concentrations of NAADP-AM used in the report by Wang *et al.* (23) are more than 3 orders of magnitude greater than those found to be effective here. However, consistent with the data presented here, these authors (23) also reported that NAADP mobilizes Ca^2+^ from acidic stores in the INS-1 β cell line, an effect blocked by Ned-19, and evoke membrane depolarization and spike generation in this cell line. We show here that NAADP-evoked Ca^2+^ transients in β cells are abolished in cells prepared from *Tpcn1*^−/−^ and *Tpcn2*^−/−^ mice (Fig. 7, *A* and *B*). We found that endogenous TPC2 proteins in human β cells co-localized with lysosomal markers (Fig. 6*B*). Interestingly, TPC2 did not appear to colocalize with insulin granules, which have also been proposed to function as NAADP-sensitive Ca^2+^ stores in β cells (13, 17).

In addition to mobilizing Ca^2+^ from acidic stores, NAADP was also found here to evoke plasma membrane cation currents and to depolarize the plasma membrane. NAADP applied through the patch pipette at low concentrations evoked a series of inward current transients (Fig. 3, *A–G*). Application of higher NAADP (100 μm) gave no response, consistent with the bell-shaped concentration-response curve for NAADP in mammalian systems and paralleling the concentration dependence of NAADP for Ca^2+^ release in β cells (Fig. 1*A*) (16, 18, 20) and other mammalian cells (58). These currents were blocked by Ned-19 and by BAPTA, suggesting that they are Ca^2+^-activated. Their abolition by replacing Na^+^ with *N*-methyl-d-glucamine suggests that the currents are cation currents largely carried by Na^+^ ions. Interestingly, a nonselective cation current has also been reported to be activated by GLP-1 (where GLP-1 is glucagon-like peptide 1), an agonist that has also been reported to elevate NAADP levels (20), in the HIT-T15 β cell line (88). The identity of the channels responsible has not been established, but our results with 9-phenanthrol may tentatively point to some involvement of the TRPM4 channels. NAADP-evoked Ca^2+^ release has recently been shown to activate TRPM4 channels in HeLa cells (65). Moreover, TRPM4 and TRPM5 channels have been proposed to mediate in part a Ca^2+^-dependent depolarization of the plasma membrane in the INS-1 β cell line (66–68, 89) and may play a general role as key components of membrane-based Ca^2+^ oscillators providing initial cell membrane depolarization for cell activation (90). The NAADP-evoked currents were found to be coincident with small NAADP-evoked Ca^2+^ transients (Fig. 7*A*), and neither NAADP-evoked Ca^2+^ transients nor currents were observed in cells from *Tpcn2*^−/−^ mice (Fig. 7*B*). We propose that these currents are due to NAADP-evoked Ca^2+^ release from endolysosomal stores via TPC2 channels and that this, in turn, via elevation of [Ca^2+^]*_i_*, leads to Ca^2+^-dependent activation of plasma membrane cation channels, possibly TRPM4 or TRPM5. Activation of these channels would then result in membrane depolarization. The finding that application of NAADP-AM elicited a series of membrane potential spikes (Fig. 4*B*) is consistent with this scenario and in agreement with a report that NAADP causes membrane depolarization in INS1 cells (23).

**FIGURE 10.**
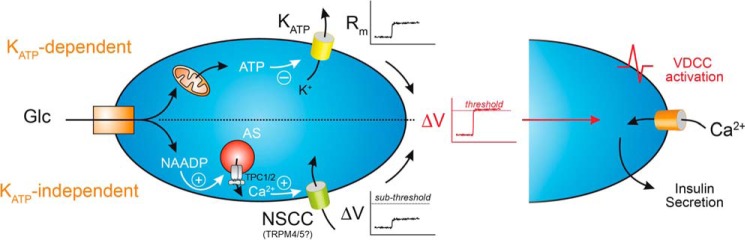
**Proposed model for synergistic effects of NAADP-regulated TPC2 and K_ATP_ channels synergizes in glucose-evoked insulin secretion.** NAADP-induced Ca^2+^ release synergizes with the K_ATP_-dependent pathway to depolarize the plasma membrane and activate VDCCs. The ATP-mediated closure of K_ATP_ channels increases membrane resistance, which together with Ca^2+^-dependent depolarizing currents (possibly TRPM4/5 channels), activated by NAADP-induced Ca^2+^-release via TPC2 expressed on acidic stores, may depolarize the plasma membrane to threshold for VDCC activation (*AS,* acidic stores; *NSCC,* nonselective cation channel; *R_m_*, membrane resistance; *VDCC,* voltage-dependent Ca^2+^ channels; *Glc,* glucose). In addition, NAADP-induced Ca^2+^-release together with VDCC-mediated Ca^2+^ influx are both required for exocytosis of insulin granules.

To investigate the role of NAADP-mediated Ca^2+^ signaling in glucose-induced electrical activity and [Ca^2+^]*_i_*, four different approaches were used to block NAADP signaling. These were as follows: (i) abrogation of Ca^2+^ storage by acidic stores with vacuolar proton pump inhibitors and GPN; (ii) inhibition of the NAADP receptor by Ned-19; (iii) self-desensitization of the NAADP receptor by NAADP; and (iv) knock-out of *Tpcn2* and *Tpcn1*, genes encoding proposed NAADP target channels. Intriguingly, high glucose was also found to evoke small Ned-19-sensitive currents similar to those evoked by pipette application of NAADP (Fig. 3*H*). Thus, NAADP signaling may contribute, at least partly, to bringing the membrane potential from rest to the threshold for activation of VDCCs (Fig. 10). As has been recognized for a long time, closure of K_ATP_ channels is not sufficient to explain how glucose depolarizes the pancreatic β cell; a depolarizing membrane current is also required (7). We propose that NAADP/TPC1/2-dependent mobilization of Ca^2+^ from an acidic intracellular store results in activation of depolarizing cation-conducting plasmalemmal ion channels and that this brings the membrane potential to the threshold for action potential firing. This is consistent with our finding that in the absence of NAADP-evoked Ca^2+^ signals in cells from *Tpcn2*^−/−^ mice, the K_ATP_ channel blocker, tolbutamide, at threshold concentrations fails to evoke Ca^2+^ signals as seen in wild-type cells (Fig. 8*I*). Indeed, it is remarkable that tolbutamide cannot by itself mimic glucose-induced Ca^2+^ signals but requires NAADP/TPCs. Indeed, tolbutamide will only evoke Ca^2+^ signals when the acidic vesicle pathway is co-stimulated either with subthreshold concentrations of NAADP/AM or with a permissive subthreshold glucose (3 mm) concentration (Fig. 8, *G* and *H*).

The final step in the stimulus-secretion coupling is the exocytosis of insulin-containing granules. In isolated islets, Ned-19 completely blocked glucose-evoked insulin secretion. Ned-19, however, had no effect on secretion evoked by depolarizing islet cells with high extracellular K^+^, which bypasses electrical activity and depolarizes the membrane potential to ∼−10 mV and opens the VDCCs. This finding makes it possible to discard the explanation that Ned-19 inhibits insulin secretion by an off-target effect on the exocytotic machinery. In a more *in vivo* setting, glucose-evoked insulin secretion from perfused whole pancreata from *Tpcn2*^−/−^ and *Tpcn1*^−/−^ mice was investigated. Insulin secretion stimulated by glucose (20 mm) was substantially reduced compared with that from wild-type animals (Fig. 9*B*). Thus, we provide evidence that NAADP signaling is an important regulator of stimulus-secretion coupling in pancreatic β cells (Fig. 10).

Surprisingly, *Tpcn*^−/−^ mice are only mildly diabetic as assessed by glucose tolerance tests (Fig. 9), with a significant impairment in *Tpcn1*^−/−^ mice. However, a recent study has implicated *TPC2* as a novel gene for diabetic traits in mice, rats, and humans (91), with a decrease in fasting glucose and insulin levels reported in *Tpcn2*^−/−^ mice. The effects of knocking out *Tpcn* genes in mice may result in complex phenotypes, including compensatory mechanisms, with regard to blood glucose and insulin levels because NAADP-mediated Ca^2+^ release has been implicated in GLUT4 translocation in murine skeletal muscle (92). Furthermore, NAADP signaling has been implicated in the action of peroxisome proliferator-activated receptor γ agonists in their insulin-sensitizing actions to ameliorate insulin resistance (93). Future studies with tissue-specific inactivation of these genes will be required to address these questions.

Although NAADP levels in β cells have been reported to be increased in response to elevated glucose, and also in response to the incretin hormone GLP-1 (18, 20), the mechanisms are not well understood. However, ADP-ribosyl cyclases have been implicated in NAADP synthesis in β cells (20). Although CD38 is a membrane-bound ecto-enzyme, glucose treatment of β cells induces endocytosis of CD38 that requires cytoskeletal changes (22). Inhibition of CD38 internalization with jasplakinolide, which promotes actin polymerization, blocks glucose-stimulated NAADP levels and impairs glucose-evoked Ca^2+^ signaling (22). NAADP synthesis is found to be associated with lysosomal membrane fractions (20). We have previously argued that cADPR (and NAADP) synthesis may occur within the acidic organelles (94). The luminal acidic pH of acidic organelles would provide an optimal environment for NAADP synthesis by ADP-ribosyl cyclases (46). We showed that pyridine nucleotides are transported into organelles, and second messenger products are transported into the cytoplasm to their site of action (94).

Antibodies to CD38 (95) and a missense mutation in the *CD38* gene (50) have been linked to type 2 diabetes, and this could potentially be accounted for by reduced NAADP synthesis. Remarkably, in a recent study, it was found that intraperitoneal injections of NAADP could restore defective insulin secretion and blood glucose regulation in *db/db* mice, an animal model of type 2 diabetes (24), presumably via the NAADP transport mechanisms described above.

## Conclusions

We propose that NAADP-evoked Ca^2+^ release from acidic stores via TPC2 or TPC1 channels evokes a small local Ca^2+^ signal that activates Ca^2+^-dependent cation currents in the plasma membrane. The finding that the membrane currents evoked by intracellular Ca^2+^ mobilization are blocked by 9-phenanthrol implicates TRPM4 channels in this process. Additional direct effects of NAADP-evoked Ca^2+^ release on exocytosis itself cannot be excluded at this stage, with a possible contribution from exocytotic granules themselves (17, 96). The NAADP/TPC/acidic organelle pathway represents a new component of the glucose-evoked trigger to depolarize the plasma membrane upon K_ATP_ channel closure resulting in VDCC-mediated Ca^2+^ influx and insulin secretion (Fig. 10). This new pathway may offer new targets for novel diabetic therapies.

## Author Contributions

A. A. and A. G. designed the experiments, and A. A. conducted the project. A. A., J. P., G. A. R., and A. G. wrote the manuscript. M. R., L. T., K. R., and J. P. produced and characterized the *Tpcn2*^−/−^ mice. F. C., T. P., and G. S. C. performed some of the [Ca^2+^]*_i_* measurement experiments. K. C. performed the gene expression experiments. R. P., A. M. L., and G. C. C. synthesized and characterized NAADP-AM, Ned-19, and Ned-20. A. J. M. designed experiments and produced Fig. 7. G. A. R. and E. A. B. performed immunocytochemical studies. K. S. performed insulin secretion experiments. P. J. supplied and prepared human islets. P. R., S. C. C., M. B., W. S., and Q. Z. performed secretion and cell physiological measurements. P. M. H. and P. W. T. performed the electron microscopy. All authors reviewed the results and approved the final version of the manuscript.

## Footnotes

5 The abbreviations used are:

VDCC: voltage-dependent Ca^2+^ channel
cADPR: cyclic adenosine diphosphate ribose
ER: endoplasmic reticulum
GPN: glycyl-l-phenylalanine-β-naphthylamide
IP_3_: inositol 1,4,5-trisphosphate
NAADP-AM: nicotinic acid adenine dinucleotide phosphate acetoxymethyl ester
TPC: two-pore channel
SERCA: sarco/endoplasmic reticulum Ca^2+^-ATPase; cytosolic Ca^2+^
NAADP: nicotinic acid adenine dinucleotide phosphate
BAPTA: 1,2-bis(2-aminophenoxy)ethane-*N*,*N*,*N*′,*N*′-tetraacetic acid.
